# The Many Faces of Sporadic Acute Q Fever, Gran Canaria: Canary Islands (Spain) (1998–2024)

**DOI:** 10.3390/pathogens15050542

**Published:** 2026-05-17

**Authors:** José-Luis Pérez-Arellano

**Affiliations:** University Institute of Biomedical and Health Research (Instituto Universitario de Investigaciones Biomédicas y Sanitarias IUIBS), University of Las Palmas de Gran Canaria (ULPGC), 35016 Las Palmas, Spain; luis.perez@ulpgc.es; Tel.: +34-928451455

**Keywords:** *Coxiella burnetii*, acute Q fever, demography, epidemiology, clinical manifestations

## Abstract

*Coxiella burnetii* is an intracellular bacterium responsible for an anthropozoonosis that can be asymptomatic or manifest as acute or chronic Q fever. This extensive series of 588 patients represents one of the largest single-center studies on sporadic acute Q fever, highlighting the Canary Islands as a high-incidence region in Spain. Epidemiologically, the domestic cycle is the primary driver of infection, with caprine livestock serving as the main reservoir, showing a local prevalence of 60.4%. Transmission is predominantly airborne via aerosols; the environmental resilience of *C. burnetii* facilitates its transport into urban areas, where the majority of patients reside despite lacking direct animal contact. While fever, headache, and diaphoresis are hallmark symptoms, over 90% of patients exhibit transient urinalysis abnormalities, a finding that often leads to misdiagnosis and inappropriate antimicrobial use. Clinically, the non-specific (45.7%) and hepatic (44.1%) forms are most prevalent, whereas the pulmonary form (7.8%) is strongly associated with smoking and alcohol consumption. Although localized forms affecting the nervous system or skin (such as panniculitis) were observed, the overall prognosis remains excellent with no progression to chronic Q fever in this series. In summary, the extensive series described characterizes acute Q fever patients in the Autonomous Community of the Canary Islands, with features that are similar in some cases but also show notable differences compared to other national and international series. Furthermore, depending on the patients’ age, the time elapsed between the onset of clinical manifestations and hospital evaluation, and the clinical form, acute Q fever displays significant differences.

## 1. Introduction

*Coxiella burnetii* is an obligate intracellular bacterium classified within the phylum Proteobacteria (gamma subdivision), the family Coxiellaceae, and the order Legionellales [1]. In addition to humans, this bacterium is capable of infecting a vast array of hosts (i.e., mammals, birds, reptiles, amphibians, fish, or arthropods) [2,3,4,5]. Although serological evidence of infection has been observed in mammals [6], they typically remain asymptomatic except during the reproductive period (i.e., miscarriages, low birth weight) [7]. Nevertheless, these animals shed *C. burnetii* in their secretions (urine, feces, milk, amniotic fluid), particularly during parturition [8]. The highest concentration of bacteria is found in milk, fetal fluids and membranes, due to its tropism for the uterus and mammary glands [9].

In humans, *Coxiella burnetii* infection can manifest in four distinct forms [1,3,10,11]: (*i*) inapparent, when there are no subjective or objective signs of disease but serological evidence of infection is confirmed; (*ii*) acute Q fever and (*iii*) chronic Q fever, characterized by subjective and objective clinical manifestations, distinguished by their temporal profile, type of symptoms and signs, and characteristic microbiological data; and (*iv*) post-Q fever, presenting with non-specific symptomatology in the absence of objective data despite microbiological evidence of infection.

Q fever exhibits a cosmopolitan distribution in both humans and other hosts [12,13]. Specifically, acute Q fever can manifest as either an autochthonous or an imported disease [14,15,16,17,18]. Within the context of acute Q fever, two distinct forms exist [8]: outbreaks (which involve a varying number of cases) [19,20,21,22,23] and sporadic cases. Furthermore, in patients with sporadic acute Q fever, the incidence, temporal pattern, clinical manifestations, and laboratory findings differ considerably between countries and, within a single country, across different regions [4,12,13,21,24,25,26].

A previous study from this line of research, published before 2000, reported the results of a brief series of patients with sporadic acute Q fever [27]. Since then, the number of observed cases has increased significantly. The two main research objectives of the present study are: (i) to provide a comprehensive clinical, epidemiological, and demographic characterization of acute Q fever in Gran Canaria over a 25-year period; and (ii) to evaluate how these clinical and epidemiological patterns vary based on three specific axes: patient age at diagnosis, the time elapsed from symptom onset to hospital evaluation, and the clinical presentation of the disease.

## 2. Patients and Methods

This was a retrospective study conducted on patients treated at the Hospital Universitario Insular de Gran Canaria (HUIGC) between 1 January 2000 and 31 December 2024. The HUIGC is one of the two primary hospitals in Gran Canaria, one of the seven largest and most populated of the Canary Islands (Spain), situated in the Atlantic Ocean between 27° and 29° North. The adult population for which the HUIGC serves as the referral hospital consists of 414,191 people, residing in the southern and eastern regions of Gran Canaria, as well as on the island of Fuerteventura.

Initially, 818 patients with at least one documented positive antibody determination against *Coxiella burnetii* phase II antigens—detected by the Microbiology Service during this period—were included, all of whom were evaluated across various departments of the HUIGC. Subsequently, medical chart data were evaluated using paper-based records up to 2011, and electronic records thereafter. Patients meeting one or more of the following conditions were excluded: (*i*) absence of clinical and/or microbiological data for proper classification (see below); (*ii*) potential imported acquisition: travelers and recent immigrants (<3 months prior to serological determination); (*iii*) diagnostic criteria for another disease (bacterial or viral infection, or neoplasia); (*iv*) immunosuppression (HIV, chemotherapy, or use of biological agents) [28,29,30,31]; (*v*) criteria for chronic Q fever [32,33] and (*vi*) post-Q fever fatigue syndrome [34].

### 2.1. Inclusion Criteria

The diagnosis of acute Q fever was based on the simultaneous presence of three criteria: (*i*) age ≥ 14 years; (*ii*) presence of clinical manifestations (compatible subjective and objective findings); and (*iii*) microbiological criteria.

The age of 14 was selected as individuals below this age are treated at another hospital within the Canary Islands healthcare area.

Regarding clinical manifestations, the definitions proposed by Raoult were employed with minor modifications [35]: (*i*) presence of data suggestive of pulmonary infection (“pulmonary”) (cough with expectoration, hemoptysis, dyspnea, or chest pain) and chest X-ray abnormalities; isolated dry cough was not considered a diagnostic criterion; (*ii*) presence of an apparent hepatic focus (“hepatic”) (elevation of ALT over two times the upper normal value), this being the limit employed by the majority of series, with some exceptions [36,37]; (*iii*) “other localized form” (i.e., nervous system, skin and soft tissues, heart, biliary tract) defined by clinical and complementary tests; and (*iv*) “non-specific” or isolated febrile syndrome, defined when fever was present without lung or hepatic involvement or another focus of infection. Some patients met the criteria for two clinical forms.

Microbiological diagnosis included three types [11,38,39]: (*i*) *defined AQF* when there was evidence of seroconversion of IgG titers to phase II *C. burnetii* (a fourfold increase or two dilutions) [7] within 2–4 weeks [4]; or positive serum PCR [40]; (*ii*) *highly probable AQF* if IgG was clearly elevated alongside positive IgM in patients for whom only a single serological determination was performed; and (*iii*) *probable AQF* when IgG serology was positive at a high titer with negative IgM, or when IgM was positive with negative IgG. Serological analysis was conducted as follows. First, a screening test was performed to determine specific IgG antibodies against *C. burnetii* phase II antigens in serum; until 2010, an indirect immunofluorescence (IFA) technique was employed (*C. Burnetii*-Spot IFA. BioMérieux, Marcy l’Etoile, France), and since then, a chemiluminescent immunoassay (CLIA) (VirClia kit, Vircell SL, Granada, Spain) has been used. Regarding IgM, the cutoff point for a positive result was ≥1:80 by IFA or ≥1:64 by CLIA, whereas for IgG, it was ≥1:128 for both techniques. High IgG titers were defined as ≥1:512. In 181 patients, the presence of antibodies against *C. burnetii* phase I antigens was also evaluated during follow-up.

### 2.2. Demographic and Epidemiological Data

Whenever available, the following *demographic* data were evaluated: age, gender, place of birth, habitat, and occupation. Regarding *age*, patients were classified into three groups: young (14–29 years old); middle-aged (30–59 years old); and older (≥60 years old) [41,42]. The terminology used in the definition of *gender* was that described by Rioux et al. [43]. Based on the patients’ *place of birth*, they were classified as Spanish or foreign, specifying the country and continent in the latter case. The *place of residence* (habitat) was categorized into three groups according to population density (inhabitants/km^2^): urban (>1500), semi-urban (300–1500), and rural (<300) [44]. Patients’ *occupation* was reported according to modified international and Spanish criteria [45,46,47,48].

*Epidemiological* data included the year and month of diagnosis, the presence of toxic habits, and exposure to potential risk factors. Regarding smoking habits, a distinction was made between current smokers, those who had never smoked, and former smokers, the latter defined as those who had ceased consumption at least three months prior to the study. Regarding alcohol consumption, participants were classified into three groups based on daily standard drink units [49]. It should be noted that the standard drink unit varies by country; in Spain, it equals 10 g of pure alcohol [50]. Risk factors included contact with animals, arthropod bites, consumption of unpasteurized dairy products (milk, cheese), and contact with wastewater. If animal contact existed, information on the primary type was recorded: domestic (dogs or cats), livestock (goats, sheep, cows), and poultry, as well as combinations thereof. The presence of arthropod bites was based on clinical history and physical examination, although in most cases, it was impossible to identify the specific type.

Furthermore, past medical history was recorded (with a particular focus on the presence of structural heart disease and/or vascular grafts) as well as the occurrence of familial cases.

### 2.3. Clinical Data

The time elapsed between the onset of clinical manifestations and evaluation (TOCE) was assessed, along with the maximum temperature reached, the prior use of antimicrobials (excluding doxycycline) and the class of drug administered, the previously indicated clinical forms of the disease, hospital admission and its duration, clinical signs and symptoms (present during the initial evaluation), and the disease progression over the six months following diagnosis (including the development of chronic Q fever, the onset of other medical conditions, and patient mortality).

### 2.4. Complementary Examinations

During the initial week of hospital evaluation, various laboratory studies were performed: complete blood count, routine coagulation tests, inflammatory biomarkers (erythrocyte sedimentation rate, C-reactive protein, and procalcitonin), serum biochemical parameters (creatinine, sodium, creatine kinase, and total γ-globulin), and urinalysis. Additionally, three sets of liver tests were measured to assess the presence and degree of cytolysis (alanine aminotransferase [ALT], aspartate aminotransferase [AST], and lactate dehydrogenase [LDH]), cholestasis (alkaline phosphatase, γ-glutamyl transferase, and direct serum bilirubin), and hepatocellular insufficiency (quick prothrombin index and serum albumin).

Furthermore, plasma concentrations of the main immunoglobulin isotypes (IgG, IgM, and IgA) were evaluated in 76 patients. Conversely, in patients whose protein electrophoresis demonstrated the presence of paraproteins, the specific type was identified via immunofixation. Additional hemostasis studies—including, among others, mixing tests, factor assays, and evaluation of antiphospholipid antibodies (lupus anticoagulant [dilute Russell’s viper venom test with confirmation using excess phospholipids], anticardiolipin antibodies [IgG and IgM], and anti-β2-glycoprotein I antibodies)—were performed in up to 59 patients, depending on previous results.

Other complementary examinations conducted included transthoracic echocardiograms (245 patients within the month following initial evaluation) and, depending on clinical manifestations, abdominal ultrasound or CT, brain or spinal CT and/or MRI, and lumbar puncture.

### 2.5. Statistics

Statistical analysis of the data obtained in the present study was performed using SPSS version 31 and Stata version 19 (StataNow).

Descriptive variables were expressed as percentages for qualitative data and as measures of central tendency and dispersion for quantitative data. Data distribution was initially assessed using the Shapiro–Wilk or Kolmogorov–Smirnov tests, as appropriate. Variables with a normal distribution were summarized as mean and standard deviation, whereas variables with a non-normal distribution were expressed as median and interquartile range.

To assess the association between three independent variables (age group, time to evaluation, and clinical group) and the dependent variables, an initial bivariate analysis was performed for exploratory purposes. Associations were evaluated using the χ^2^ test, and when an association was identified, Cramér’s V was calculated to assess its strength. Subsequently, multivariate analysis was conducted using logistic regression (ordinal or binary, depending on the type of dependent variable) and multinomial logistic regression for nominal polytomous variables, with the aim of identifying independent associations. The results of the multivariate analysis were expressed as odds ratios (ORs) with 95% confidence intervals (CIs). Statistical significance was defined as a two-tailed *p* value < 0.05.

### 2.6. Ethics

The present study was conducted in accordance with the guidelines of the 1975 Declaration of Helsinki, as revised in 2013. The protocol was approved by the Research Ethics Committee/Drug Research Ethics Committee (CEI/CEIm) of Las Palmas (Canary Islands, Spain) (Approval Code: 2025-209-1; Approval Date: 24 April 2025). Given the retrospective nature of the study and the use of anonymized clinical data, the requirement for individual informed consent was waived [51].

## 3. Results

**Figure 1:**
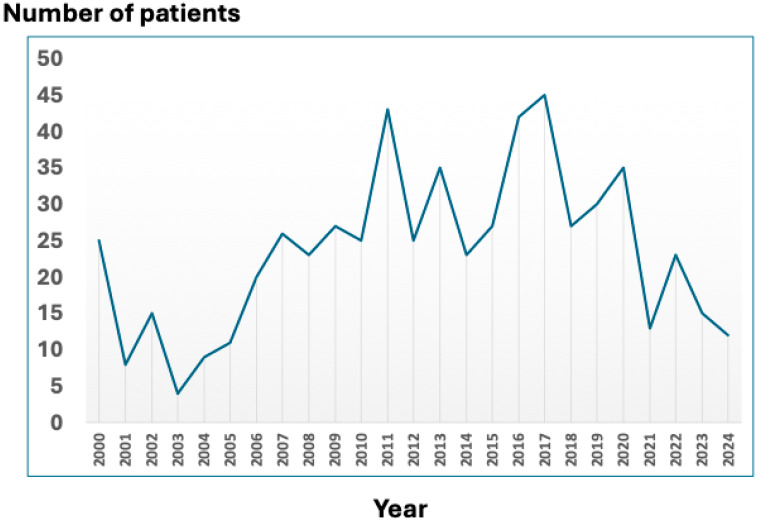
Annual Trend in Patient Numbers.

During the study period, 588 patients were registered over 25 years, with an annual mean of 23 cases (Figure 1). Graphical analysis using a 5-year moving average revealed a non-strictly linear pattern, characterized by an initial low-frequency phase during the early years of the registry, a sustained increase with peaks between 2011 and 2017, and a relative decline in the most recent years of the analyzed period.

The main demographic data are shown in Table 1 and Table S1. Regarding gender, two cisgender women were pregnant at the time of illness. Furthermore, a significant percentage of patients were born in countries other than Spain. When this data was compared with the general population of Gran Canaria, significant differences were observed (*p* < 0.05), with a higher prevalence among foreign nationals. Specifically, the proportion was higher among those born in the Americas (particularly Cuba, Colombia, and Venezuela) and lower among those originating from Eurasia, Africa, or Asia.

The main epidemiological data are detailed in Table 2.

In 225 patients (38.3%), there were no relevant medical antecedents, while in the remaining cases, one or several common medical conditions were detected. These primarily included hypertension, diabetes mellitus, dyslipidemia, COPD, and gastroesophageal reflux. Surgical history was also frequently noted, particularly appendectomy or tonsillectomy. Additionally, a history of cardiac and vascular disorders was common (especially ischemic heart disease or arrhythmias; however, only three patients had a history of valvular lesions), and none had vascular grafts. No familial cases of Q fever were documented.

Table 3 provides details on the primary general clinical data for this series.

Regarding the type of antibacterials used, 61% were beta-lactams (primarily amoxicillin/clavulanate), 28% fluoroquinolones (mainly ciprofloxacin), and 24% macrolides (specifically azithromycin). Table S2 shows the main localized syndromes, excluding liver and lung involvement. The symptoms and signs present during the initial evaluation are indicated in Table 4. Figure S2 displays the percentage of patients with acute Q fever who were hospitalized, categorized by the year of the study.

In 181 of the 588 patients (30.8%) with acute Q fever, a 6-month follow-up was completed. Although this limited follow-up rate could potentially affect the documented incidence of chronic Q fever, no clinical consultations regarding subsequent related complications were recorded among the lost-to-follow-up patients within the HUIGC database. 15 patients presented IgG antibodies against phase I (with the following titers: 1:1024 [7], 1:2048 [5], 1:4096 [1], 1:8192 [1], and 1:16,384 [1]); none of them met other criteria for chronic Q fever. During this period, several new medical problems were observed, both neoplastic (two B-cell lymphomas, two lung adenocarcinomas, one gastric adenocarcinoma, one laryngeal neoplasm, one cancer of unknown primary with multiple metastases, and one Warthin’s tumor) and of other origins (basal ganglia calcification and a colloid cyst of the third ventricle). During the six-month follow-up after diagnosis, only one patient died from liver failure attributable to the infection.

Tables S3 and S4 present the main findings from the complementary examinations.

Among the 76 patients in whom the serum concentrations of the main immunoglobulin isotypes (IgG, IgM, and IgA) were quantified, highly variable results were found: levels were within the normal range in 32 cases and elevated for all three isotypes in 3 cases. In 4 cases, an elevation of two different isotypes was detected (1 IgG + IgA, 3 IgM + IgA), while in the remaining patients, alterations in a single isotype were observed (elevated IgG in 1 patient and decreased in 2; elevated IgM in 17 patients; and elevated IgA in 17 cases and decreased in 1).

In 17 patients, alterations in the protein electrophoresis (proteinogram) other than hypergammaglobulinemia and hypoalbuminemia were observed. In 15 patients, these corresponded to paraproteins (double in 3 cases; single in 12 [8 IgG, 2 kappa/5 lambda; 4 IgM, 2 kappa/5 lambda]). The remaining two cases corresponded to IgG biclonality and a beta-gamma bridge.

The main alteration observed in the complementary coagulation studies was the presence of antiphospholipid antibodies (24 patients), especially lupus anticoagulant (17), anticardiolipin (4), and both (2). Other less frequent findings were contact phase alteration (8), factor deficiency (1), and disseminated intravascular coagulation (1).

A very important aspect that should be noted is that most of the clinical and analytical data were observed early during the acute phase and were transient in nature, disappearing during patient follow-up.

Of the 245 transthoracic echocardiograms performed, valvular lesions confirmed by TTE were observed in only 12 of them.

In the bivariate analysis of the association between the age group and the remaining variables (Table S5a), a statistically significant association was observed with demo-epidemiological data (place of birth, habitat, tobacco use, and animal contact) and clinical-analytical data (time elapsed between the onset of clinical manifestations and evaluation, hospital admission, serum ALT and GGT levels, diagnostic group, and clinical group). When the independent association of variables was evaluated using the 30 to 59 years group as the reference value, several associations were observed as indicated in Table S5b.

When the bivariate analysis of the association of time to hospital evaluation with the rest of the variables was performed (Table S6a), no statistically significant association was observed with demo-epidemiological data; however, associations were found with clinical-analytical data (platelet count, aPTT ratio, ESR, CK, ALT, LDH, GGT, diagnostic, and clinical group). Multivariate analysis, using the 8–21 group as the reference value, showed several associations indicated in Table S6b.

In the bivariate study of the association of the clinical group with the remaining variables (Table S7a), a statistically significant association was observed with demo-epidemiological data (gender, tobacco, or alcohol use) and clinical-analytical data (time elapsed between the onset of clinical manifestations and evaluation, hospital admission, arthralgias, headache, myalgias, white blood cell count, platelet count, C-reactive protein, ALT, LDH, GGT, overall urinalysis, and diagnostic group). In the multivariate study, using the “non-specific” group as the reference value, several associations were observed as indicated in Table S7b.

## 4. Discussion

Including 588 patients with *sporadic* acute Q fever, this study represents, to our knowledge, one of the largest and longest single-center series reported [5,11,21,35,36,37,52,53,54,55,56,57,58,59,60,61], even when compared with cohorts focusing solely on pulmonary or hepatic forms [62,63]. The analysis of the results and, specifically, the comparison with the other aforementioned series, is difficult due to variation in methods of case finding, diagnostic testing, reporting, and surveillance data [58].

The *number of cases* in this series, similar to others previously published, exhibits significant annual variations, with mean values exceeding those reported in the literature for other Spanish regions [5,21,26,36,53,54,55,56,57] with few exceptions [11]. In recent years, an apparent increase in the incidence of this disease has been observed, which may be due to increased reporting or diagnosis [6,35]. The actual incidence of Q fever in Canary Islands is likely underestimated, as cases managed exclusively in primary care or private centers were not included [9,11]. Although Q fever has been a notifiable disease in Spain since 2015 (Centro Nacional de Epidemiología [National Epidemiology Centre]), indirect data (comparing notifications with hospitalizations) suggests the existence of underreporting [58]. Despite these limitations, the Canary Islands is the community with the highest incidence of notification and hospitalization (3.95 and 2.95 per 100,000 inhabitants/year) [58].

Regarding *gender*, the majority of patients are male, as is the rule in all series to a greater or lesser extent. Although occupation and/or contact with animals may play a role in certain cases, the most plausible explanation based on experimental studies is the protective role of estrogens (i.e., 17 ß estradiol) [10].

Considering *age* at the time of diagnosis, the majority of cases, both in the current series and in most other published works, correspond to middle-aged patients [24]. Nevertheless, up to 30% of them were younger (20%) or older (10%). In the multivariate study, the 14–29 age group was less likely to have a foreign origin, an urban habitat, current or previous tobacco consumption, or elevated GGT levels; conversely, a shorter interval between time to evaluation and confirmed diagnosis was more frequent. On the other hand, in the age group ≥60 years, hospital admission, a very likely diagnosis, and non-specific and pulmonary forms were more frequent, while elevated ALT levels were less common.

We have found no references in the literature regarding other demographic aspects or risk factors such as *place of birth*, nor the (current or previous) *consumption of tobacco or alcohol*. In this study, when the origin of the patients was compared with the general population, an increase in individuals born outside of Spain was observed, particularly from Latin America. The reason for this finding remains speculative, potentially due to greater exposure or a specific genetic predisposition, though no data exist to support this. On the other hand, investigating the relationship with tobacco consumption was of interest, given that other granulomatous diseases (i.e., sarcoidosis or hypersensitivity pneumonitis) are less frequent in smokers [64]. Although the study design precluded a definitive evaluation of this aspect, it was observed that tobacco consumption was more frequently associated with pulmonary forms. An association between excessive alcohol consumption and pulmonary forms was also observed.

No *familial clustering* of Q fever patients was observed in this series. Reports of acute familial Q fever are anecdotal [36,65,66,67] and have been linked to shared contact during the birthing of infected pets or contaminated work clothing (“second-hand Q fever”).

Regarding the *epidemiology* of acute Q fever in Gran Canaria, as in other parts of the world, the domestic/peridomestic transmission cycle of *C. burnetii* predominates [8,13]. The three basic elements of this cycle are the reservoir or source of infection, the mode of transmission, and host characteristics. The primary reservoir comprises livestock (especially ruminants such as goats, sheep, and cattle) and, to a lesser extent, companion animals (dogs and cats) [9,68]. Thus, in a seroepidemiological study of a representative sample of ruminants in Gran Canaria, 60.4%, 31.7%, and 12.2% of goats, sheep, and cattle, respectively, were found to be infected [69], this being one of the Canary Islands with the largest livestock populations (including semi-intensive farms and feral goats).

The primary mode of transmission is the airborne route through aerosols containing infectious bacteria, although other forms have been described, such as the consumption of contaminated milk or its derivatives, and other less frequent routes [7,13]. Other factors, such as the consumption of unpasteurized dairy products, exposure to wastewater, or tick bites, appear to play a less relevant role in this series. In this series, several data suggest a significant role for animals, such as the high percentage of patients reporting contact with them (approximately 70%), the previously indicated high prevalence of infection in livestock in Gran Canaria, and a monthly distribution that coincides with the kidding (birthing) season of goats. Regarding animal contact, data from different series are highly diverse, ranging between 2% and 83% of patients [5,11,21,35,36,52,53,54,61]. However, other data from our series limit the importance of direct animal contact. Thus, occupational risk exposure (including agriculture, livestock, and environmental management or healthcare and social services) accounts for only 8% of patients. On the other hand, although the exact definition of rural and urban habitat is detailed in very few series [36,53], the percentage of patients with urban residence is very high, often exceeding rural cases [11,26,35,53,54,55,56,57]. A unifying explanation for all these data is based on the resistance of *C. burnetii* to adverse environmental conditions, allowing it to be transported many kilometers away while maintaining great virulence from its origin. Environmental conditions (wind, humidity, and temperature) and other factors (i.e., the use of face masks during the COVID-19 pandemic) could all affect the variation in annual cases. Finally, host genetic factors (e.g., HLA-DRB1*04 was more frequent in patients compared to the control group) may explain why a microorganism transmitted via the airborne route does not cause disease in all infected individuals [70].

It is generally assumed that the incubation period of *C. burnetii* infection in humans is 18 days (95% CI 7-32) [71]. However, this figure is difficult or impossible to ascertain concretely in individual cases. Although data in the literature are scarce, an aspect of interest is the *time elapsed between the onset of clinical manifestations and hospital evaluation*. In the present series, this global figure is 8 (5–14) days, which is similar to or slightly lower than those reported in other studies. [11,26,37,53]. In the majority of patients, the TOCE (Time to Clinical Evaluation) was ≤7 days (short duration) or between 8 and 21 days (intermediate duration), being much less frequent beyond 21 days (long duration). It is worth noting that although acute Q fever is a common cause of “fever of intermediate duration”, practically half of Q fever cases are of short duration. In the multivariate analysis, the short duration group was less likely to present a prolonged aPTTr (activated partial thromboplastin time ratio) or increased ESR (erythrocyte sedimentation rate) or GGT; conversely, thrombopenia and elevated CK (creatine kinase) were more frequent. On the other hand, in the long duration group, elevated LDH or ALT was less likely, while thrombocytosis and non-specific or localized clinical forms were more frequent.

Approximately one-fourth of patients with acute Q fever *required hospital admission*, a percentage lower than that reported in other published series [11,21,53,54,58,61]. An aspect that warrants highlighting is the discrepancy between the annual reduction in case incidence and the number of admissions. One interpretation for this finding is that an increased awareness of the disease ensures that only the most severe cases are referred to the hospital.

In this series, the most frequent *clinical manifestations* were fever, headache, arthralgia, and myalgia. High-grade fever was observed in 97.4% of patients, frequently accompanied by profuse diaphoresis (70%). These data are consistent with most published series [36,37,53,54,57] although it is lower in a small percentage of them [5,11,21,55,72]. The presence of headache was also frequent (7 out of 10 patients), higher than in other series [5,11,35,37,53,56,58] and lower than others [26,54,61]. Just over half of the patients presented with arthralgia and/or myalgia, which is difficult to compare with other series as they do not specifically differentiate between both types of symptoms. Laboratory data evaluation indicates two aspects of interest. Firstly, the detection of inflammatory biomarkers is very frequent, especially C-reactive protein and, to a lesser extent, ESR. Secondly, more than 90% of the patients in this series presented one or more abnormalities in urinalysis, in the absence of urinary casts or glomerular function impairment. To our knowledge, urinalysis alterations have only been noted in one previous publication [37]. Although it is very difficult to evaluate the mechanisms behind these alterations due to their transient nature, their presence is of practical interest. In this sense, they may be related to the inappropriate use of antimicrobials when interpreted as a surrogate marker of urinary tract infection.

The most frequent *clinical forms* in Gran Canaria are the non-specific type, accounting for 45.7% of the total, and the hepatic form (either isolated or associated), present in 44.1% of patients. The pulmonary form is infrequent, whether isolated or associated, with an incidence of 7.8%. Other localized forms account for 7.4% of cases in this series. An aspect to highlight in this study is the high incidence of localized forms other than the liver or lung, which is higher than in most other published series. The majority corresponded to involvement of the nervous system and, in descending order, skin and soft tissue, biliary tract, and heart. Several of the neurological forms have already been described by other authors, such as meningoencephalitis [73,74], ischemic stroke [75,76], transverse myelitis [76,77], and optic neuritis [78,79]; however, we have found no references regarding the association of Q fever with cerebral venous sinus thrombosis, hearing loss, or dysgeusia. Specific skin lesions in acute Q fever are infrequent (6.3% in this series). Nevertheless, we have also observed other better-defined skin and soft tissue lesions, primarily in the form of panniculitis (septal or lobular) without vasculitis, as previously described by other authors [80,81,82,83]. Furthermore, several patients presented other manifestations such as Sweet syndrome, lichenoid pityriasis, or angioedema, which, to our knowledge, have not been reported in association with acute Q fever. In this series, the most frequent form of biliary tract involvement was cholangitis, unlike other published cases where cholecystitis was observed [84,85]. Unlike chronic Q fever, in which endocarditis is characteristic, cardiac involvement in acute Q fever in this series manifests as pericardial or myocardial lesions, as previously reported [86,87,88,89].

Literature analysis indicates different *clinical patterns* depending on the geographical area. Thus, for instance, in Spain, non-specific forms predominate in Extremadura [36] or the Balearic Islands [11]; hepatic forms in Andalucia [26], Castilla-La Mancha [53], and the Valencian Community [54]; and pulmonary forms in the Basque Country [62] or Galicia [21]. The distinct clinical expression of acute Q fever in the aforementioned geographical areas appears to depend, among other factors, on the size of the inoculum, the route of infection acquisition, and the virulence and genotype of the *C. burnetii* strain [90,91,92].

Finally, it should be noted that the clinical forms indicated are independently associated with various distinct epidemiological and clinical data. Specifically, in the *hepatic form*, a shorter time to hospital evaluation, the presence of hematological alterations (thrombopenia and leukopenia), marked alteration of GGT and LDH enzymes, an elevation of C-reactive protein, abnormalities in urinalysis, and a higher frequency of definitive cases were more frequent. On the other hand, in the *pulmonary form*, tobacco consumption (current or previous) and excessive alcohol consumption, hospital admission, hematological alterations such as leukocytosis or thrombo-cytosis, and “highly probable” diagnosed cases were more frequent. Finally, in the group of *other localized forms*, hospital admission, leukocytosis, increased GGT, and “highly probable” diagnosed cases were more common.

In the follow-up of patients with acute Q fever, death is exceptional in this series, as is the case in virtually all other published works. Furthermore, despite limitations due to the small number of patients who attended follow-up visits, none of them presented criteria for chronic Q fever, regardless of the data indicated over the years as predictors of this progression, such as advanced age, previous valvular involvement, Phase I antibody titers against *C. burnetii*, prolongation of aPTTr, or the detection of anticardiolipin antibodies [57,93,94]. On the other hand, other diseases were observed during this follow-up, especially neoplasms, some of which have already been previously noted, such as lymphomas or carcinomas [95,96,97,98,99,100], although establishing a causal relationship with *C. burnetii* infection remains very difficult.

This study has several *limitations* that should be acknowledged, primarily stemming from its retrospective and single-center design. This nature restricted the data to that collected as part of routine clinical surveillance, which may limit the availability and precision of information regarding specific environmental exposures and clinical details, particularly in older cases where standardized data collection protocols were not yet established. Furthermore, because this is a hospital-based series, the findings are limited to patients diagnosed within this setting. This may introduce a selection bias toward more severe clinical cases and underestimate total incidence, as milder episodes managed in primary care or private clinics were not captured. Additionally, the transient nature of clinical and laboratory alterations, such as transaminases or C-reactive protein, may introduce analytical discrepancies depending on the timing of evaluation; however, we attempted to mitigate this by selecting determinations closest to the clinical event. Finally, a diagnostic bias cannot be ruled out, as episodes of acute Q fever—especially those presenting as isolated pneumonia or acute hepatitis—may have gone unnoticed since serological testing is not routinely performed in these clinical situations unless a fever is prolonged and without an evident source.

## 5. Conclusions

In summary, the extensive series described characterizes acute Q fever patients in the Autonomous Community of the Canary Islands, with features that are similar in some cases but also show notable differences compared to other national and international series. Furthermore, depending on the patients’ age, the time elapsed between the onset of clinical manifestations and hospital evaluation, and the clinical form, acute Q fever displays significant differences.

## Figures and Tables

**Table 1:** Demographic Data.

Variable (Available Data)	Category	n	%
Age (N = 588)	14–29	118	20.1
	30–59	411	69.9
	≥60	59	10.0
Gender (N = 588)	Cisgender men	492	83.7
	Cisgender women	96	16.3
Place of birth (N = 588)	Spain	482	82.0
	Other countries	106	18.0
Habitat * (N = 576)	Urban	166	28.8
	Semi-urban	319	55.4
	Rural	91	15.8
Occupation (N = 463)	Paid employment	353	76.2
	Unpaid work	44	9.5
	Not established	66	14.3

* Habitat classification based on population density and local administrative criteria.

**Table 2:** Epidemiological Data.

Variable (Available Data)	Characteristics	n	%
Tobacco use (N = 484)	Current smoker	199	41.1
	Never smoker	222	45.9
	Former smoker	63	13.0
Alcohol use (N = 447)	Low or no consumption	253	56.6
	Moderate consumption	174	38.9
	Excessive or heavy consumption	20	4.5
Animal contact (N = 458)	No	151	33.0
	Yes (Total)	307	67.0
	*Domestic animals* (*dogs*, *cats*)	171	*37.3*
	*Livestock* (*goats*, *sheep*)	40	*8.7*
	*Domestic animals and livestock*	32	*7.0*
	*Birds*	26	*5.7*
	*Birds and domestic animals*	16	*3.5*
	*Birds and livestock*	4	*0.9*
	*Other animals*	7	*1.5*
	*Mixed animal groups*	11	*2.4*
Arthropod bites (N = 588)	No	539	91.7
	Yes	49	8.3
Consumption of unpasteurized dairy (N = 276)	No	227	82.2
	Yes	49	17.8
Exposure to wastewater (N = 249)	No	221	88.8
	Yes	28	11.2

**Table 3:** General clinical data.

Variable (Available Data)	Median (IQR)	Category	Number	%
TOCE * (days) (N = 554)	8 (5–14)	≤7	266	48.0
		8–21	248	45.0
		≥22	40	7.0
Maximum body temperature (°C) (N = 581)	39.4 (39.0–40.0)	<37.5	11	2.2
		37.5–37.9	4	0.8
		38.0–38.4	44	8.8
		38.5–38.9	50	10.0
		39.0–39.4	152	30.3
		39.5–39.9	86	17.2
		>40	154	30.7
Pre-hospital antimicrobial use (N = 473) **		No	302	63.8
		Yes	171	36.2
Hospital admission (see Figure S2) (N =588)		No	450	76.5
		Yes ***	138	23.5
Clinical group (see Table S2) (n = 588)		Hepatic only	234	39.8
		Hepatic + Pulmonary	10	1.7
		Hepatic + OLF ****	15	2.6
		Pulmonary only	32	5.4
		Pulmonary + OLF ****	4	0.7
		OLF ****	24	4.1
		Non-specific	269	45.7

* TOCE = time elapsed between the onset of clinical manifestations and evaluation. ** Excluding doxycycline. *** Median length of hospital stay (days), expressed as median and interquartile range = 22 (16–31). **** OLF = Other localized forms including the central nervous system, skin and soft tissues, biliary tract, and heart.

**Table 4:** Clinical symptoms and signs.

Variable (Available Data)	Category	Number	%
Abdominal pain (N = 452)	Present	112	24.8
	Absent	340	75.2
Arthralgias * (N = 343)	Present	203	59.2
	Absent	140	40.8
Conjunctivitis (N = 368)	Present	29	4.9
	Absent	339	92.1
Nonproductive cough (N = 478)	Present	157	32.8
	Absent	321	67.2
Cutaneous rash ** (N = 558)	Present	35	6.3
	Absent	523	93.7
Profuse diaphoresis (N = 337)	Present	237	70.3
	Absent	100	26.7
Diarrhea (N = 569)	Present	66	11.6
	Absent	503	88.4
Headache (N = 441)	Present	322	73.0
	Absent	119	37.0
Heart murmur (N = 556)	Present	15	2.7
	Absent	541	97.3
Hepatomegaly (N = 558)	Present	71	12.7
	Absent	487	87.3
Jaundice (N = 551)	Present	19	3.4
	Absent	532	96.6
Lymphadenopathy (N = 549)	Present	21	3.8
	Absent	528	96.2
Altered mental status ** (N = 454)	Present	24	5.0
	Absent	430	95.0
Myalgias (N = 385)	Present	235	61.0
	Absent	150	39.0
Nausea and/or vomiting (N = 460)	Present	167	36.3
	Absent	293	63.7
Odynophagia (N = 387)	Present	43	11.1
	Absent	344	88.9
Pharyngitis (N = 552)	Present	14	2.5
	Absent	538	97.5
Splenomegaly (N = 559)	Present	31	5.5
	Absent	528	94.5

* Arthritis was documented in five patients. ** Excluding specific skin lesions.

## Data Availability

Author has full access to and is the guarantor for the data. The datasets generated are available from the corresponding author upon reasonable request.

## Supplementary Materials

The following supporting information can be downloaded at: https://www.mdpi.com/article/10.3390/pathogens15050542/s1, Figure S1. Monthly distribution of acute Q fever; Figure S2. Hospital admission; Table S1. Occupation; Table S2. Other localized syndromes; Table S3. Laboratory Test (I); Table S4. Laboratory Test (II). Liver tests; Table S5a. Bivariate analysis of associations with age group; Table S5b. Multivariate analysis of associations with age group; Table S6a. Bivariate analysis of associations with time to clinical evaluation; Table S6b. Multivariate analysis of associations with time to clinical evaluation; Table S7a. Bivariate analysis of associations with clinical group; Table S7b. Multivariate analysis of associations with clinical group.
