# Supplementary material for: The Many Faces of Sporadic Acute Q Fever, Gran Canaria: Canary Islands (Spain) (1998–2024)
Source: Pathogens. 2026 May 17;15(5):542. doi: 10.3390/pathogens15050542 (PMC13209193; doi:10.3390/pathogens15050542)
Supplement: Supplementary file 1 [file pathogens-15-00542-s001.zip › pathogens-4303004-supplementary.pdf]

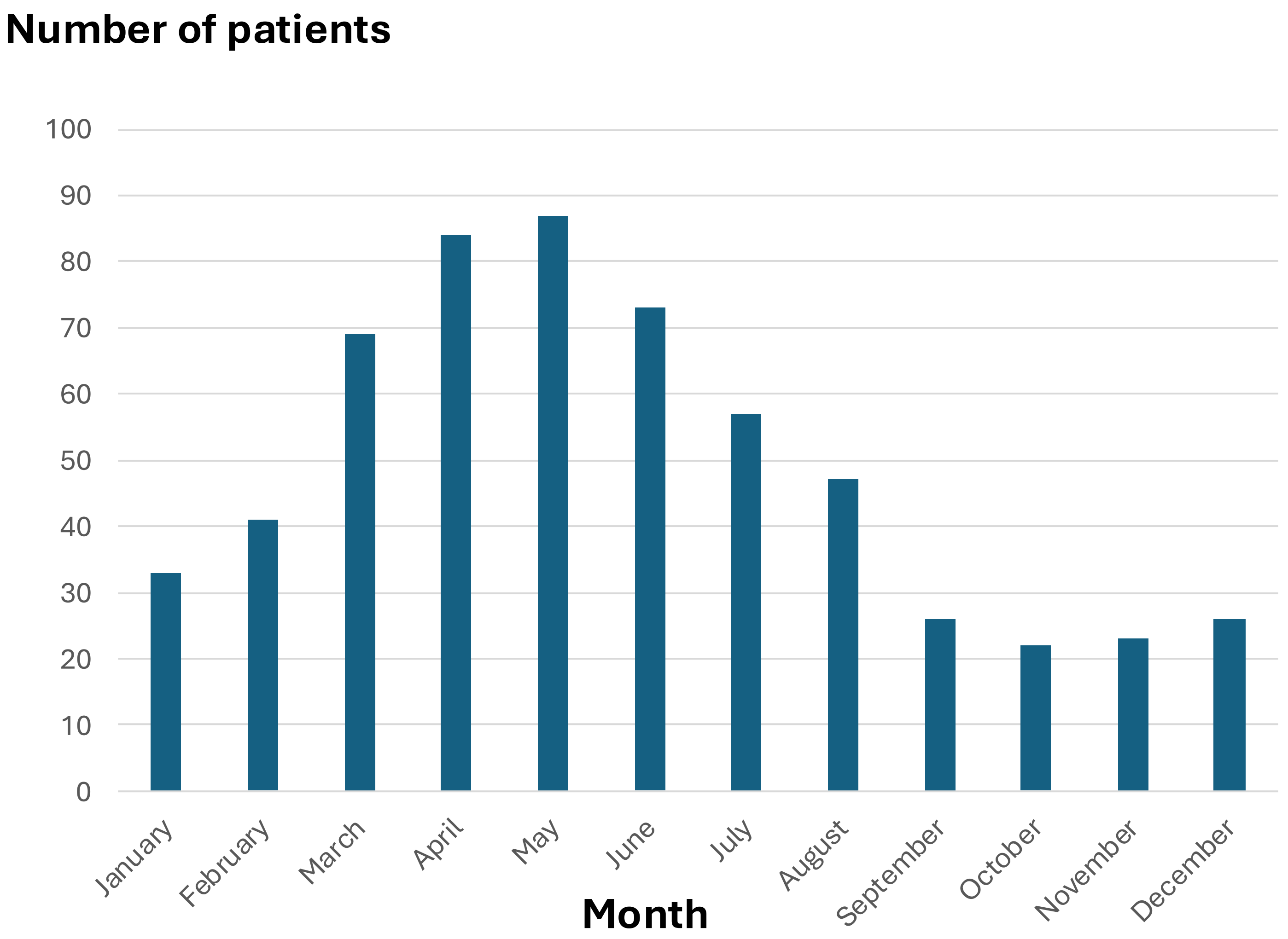

Figure S1. Monthly distribution of acute Q fever

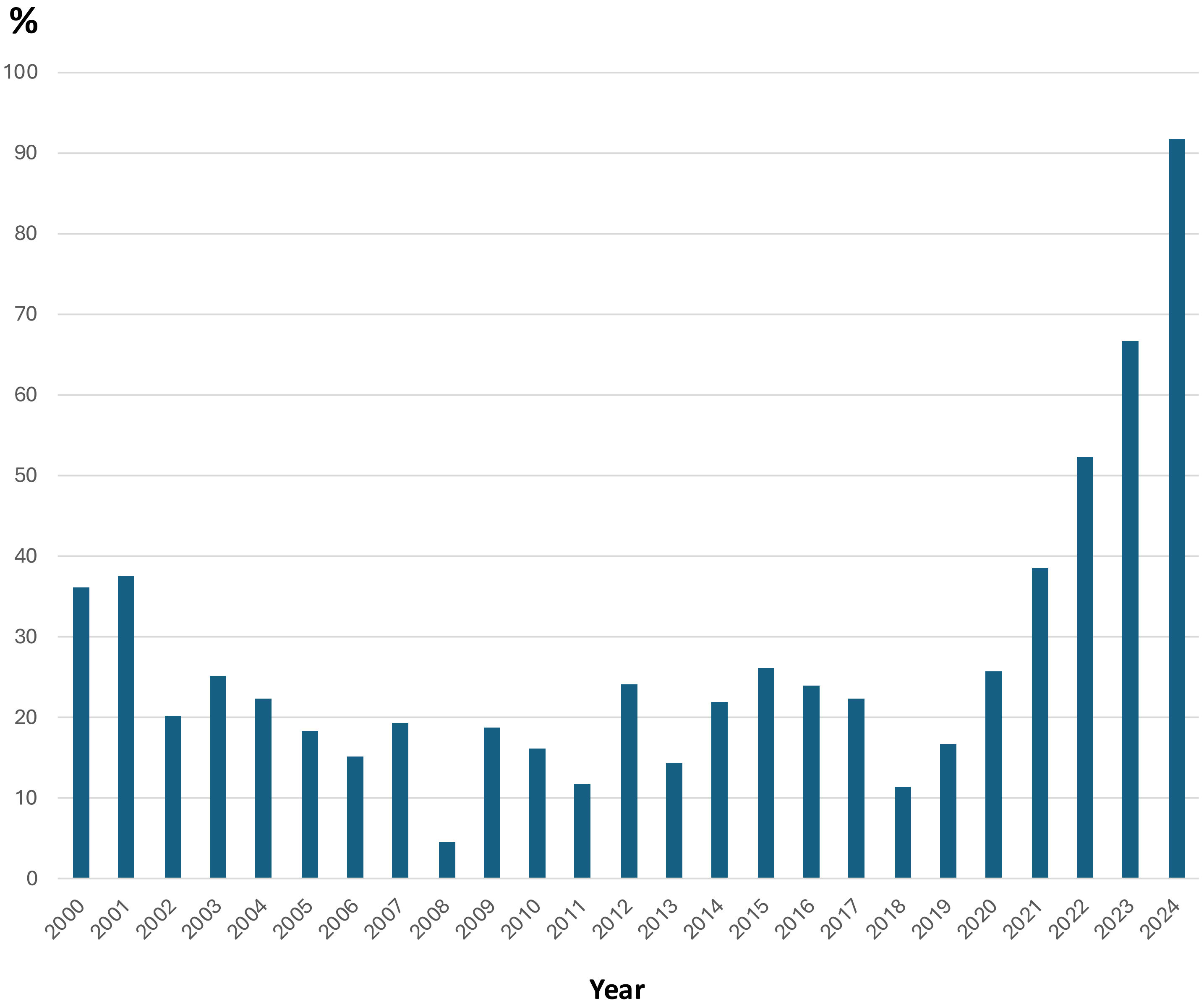

Figure S2. Hospital admission

Table S1. Occupation

Category	n	%
**Not Specified**	**66**	**14.25**
Retirement/Disability	33	7.13
Unemployment	32	6.91
Extreme poverty / Indigence	1	0.22
**Unpaid work**	**44**	**9.50**
Homemakers	14	3.02
Students	30	6.48
**Paid employment**	**353**	**76.24**
Agriculture, livestock, and environmental management	17	3.67
Arts, culture, and communication	7	1.51
Commerce, sales, and customer service	30	6.48
Construction and building trades	45	9.72
Education and professional training	11	2.38
Healthcare and social services	20	4.32
Hospitality, food service, and catering	61	13.17
Industrial operations, maintenance, and technical trades	29	6.26
Information technology and digital systems	8	1.73
Logistics, warehousing, and distribution	13	2.81
Public administration and administrative services	33	7.13
Personal and community services	17	3.67
Security, emergency services, and law enforcement	10	2.16
Tourism, leisure, and related services	11	2.38
Transportation and professional driving	41	8.86

Table S2. Other localized syndromes*

Localization	Total	Syndrome	Number
**Central nervous system**	**24**	Meningoencephalitis	10
		Ischemic stroke	7
		Myelitis	2
		Optic neuritis	2
		Cerebral venous sinus thrombosis	1
		Hearing loss	1
		Dysgeusia	1
**Skin and soft tissue**	**13**	Septal panniculitis without vasculitis	7
		Lobular panniculitis without vasculitis	2
		Sweet syndrome	2
		Lichenoides pityriasis	1
		Angioedema	1
**Biliary tract**	**4**	Cholangitis	4
**Heart**	**3**	Dilated cardiomyopathy	1
		Acute myopericarditis	1
		Pericardial effusion	1
**Other**	**2**	Pulmonary thromboembolism	1
		Spleen infarction	1

* Excluding isolated hepatic or pulmonary involvement.

Table S3. Laboratory Test (I)

Variable (Available data)	Category	Abnormal values	Number (n)	%	Median (IQR)*
**Complete Blood Count**	**Hemoglobin (g/dL)** (n = 574)	Anemia	94	16.4	11.5 (11.3–11.7)
	**Mean corpuscular volume (fL)** (n = 570)	Microcytosis	19	3.3	74.8 (72.8–76.9)
		Macrocytosis	4	0.7	105 (101–109)
	**White blood cell count x 10³µL)** (n = 579)	Leukopenia	42	7.2	3.4 (3.2–3.6)
		Leukocytosis	91	15.8	14.6 (14.0–15.2)
	**Platelet count (x 10³µL)** (n = 577)	Thrombocytopenia	129	22.3	109 (105–112)
		Thrombocytosis	42	7.3	501 (479–524)
**Coagulation studies****	**aPTT ratio** (n = 425)	Decreased	8	1.9	0.7 (0.6–0.8)
		Increased	154	36.2	1.5 (1.3–1.7)
**Inflammatory biomarkers**	**Erythrocyte sedimentation rate (mm/h)** (n = 358)	Elevated	243	67.9	34 (20–52)
	**C-reactive protein (mg/dL)** (n = 280)	Elevated	222	79.3	8.9 (3.3–14.9)
	**Procalcitonin (ng/mL)** (n = 162)	Elevated	88	54.3	1.18 (0.6–1.9)
**Serum biochemical parameters**	**Serum creatinine (mg/dL)** (n = 566)	Decreased	60	10.6	0.63 (0.57–0.66)
		Elevated	54	9.6	1.37 (1.3–1.5)
	**Serum sodium (mmol/L)** (n = 550)	Decreased	243	44.2	133 (131–134)
		Elevated	3	0.5	147 (146-151)
	**Creatine kinase (U/L)** (n = 311)	Decreased	27	8.7	15 (11–18)
		Elevated	32	10.3	419 (290–544)
	**Serum γ–gamma-globulin (g/dL)** (n = 88)	Elevated	19	21.6	2 (1.6–2.1)
**Urinalysis**	**Overall urinalysis** (n = 394)	Altered	325	92.5	-
	**Microhematuria** (n = 390)	Positive	234	60.0	-

**Leukocyturia** (n = 393)	Positive	199	50.6	-
**Proteinuria** (n = 394)	Positive	258	65.5	-

* IQR: Interquartile range.

** Abnormal hemoglobin value: Defined as < 13 g/dL in males and < 12 g/dL in females.

*** Additional data: Provided in **Table 6** and in the main text.

Table S4. Laboratory Test (II). Liver tests

Variable (Available data)	Abnormal values	Number (n)	%	Median (IQR)*
**Cytolisis**				
**AST (U/L)** (n = 558)	Elevated	497	89.1	148 (87–241)
**ALT (U/L)** (n = 557)	Elevated	496	89.1	165 (93–296)
**LDH (U/L)** (n = 398)	Elevated	335	84.2	344 (275–471)
**Cholestasis**				
**GGT (U/L)** (n = 496)	Elevated	425	85.7	196 (105–349)
**Alkaline phosphatase (U/L)** (n = 533)	Elevated	400	75.0	198 (139–304)
**Total bilirubin (mg/dL)** (n = 551)	Elevated	58	10.5	1.6 (1.3–2.6)
**Hepatocellular insufficiency**				
**Serum albumin (g/dL)** (n = 432)	Decreased	321	74.3	2.9 (2.6–3.2)
**Quick ratio (%)** (n = 417)	Decreased	42	10.1	62 (52-27)

* IQR: Interquartile range.
** ULN: Upper limit of normal.
*** In 35 patients, the ratio of direct to total bilirubin exceeded 30%.

Table S5a. Bivariate analysis of associations with age group*

Variable	$\chi 2$	df**	p	Cramer's V
Place of birth	8.38	2	0.015	0.12
Habitat	15.11	4	0.019	0.11
Tobacco use	39.30	4	< 0.001	0.20
Animal contact	12.47	2	0.002	0.17
Time to hospital evaluation	12.39	4	0.015	0.11
Hospital admission	10.80	2	< 0.001	0.14
ALT***	16.81	6	0.010	0.12
GGT***	14.33	2	< 0.001	0.17
Diagnostic group	15.11	4	< 0.001	0.11
Clinical group	28.50	6	< 0.001	0.16

* Only variables with a statistically significant association are included.
** df: degrees of freedom.
*** ALT:alanine aminotransferase; GGT:gamma-glutamyl transferase.

Table S5b. Multivariate analysis of associations with age group*

Variable	Regression model	Comparison**	OR***	95% CI***	p value
**Place of birth**	Logistic				
Foreign-born vs spanish		14-29 years	0.49	0.26–0.94	0.033
**Habitat**	Multinomial				
Semi-urban vs urban		14-29 years	2.49	1.44–4.31	0.001
**Tobacco use**	Multinomial				
Former smoker vs non-smoker		14-29 years	0.11	0.02–0.45	0.002
Former smoker vs non-smoker		≥60 years	2.27	1.08–4.77	0.030
Smoker vs non-smoker		14-29 years	0.59	0.37–0.94	0.025
Smoker vs non-smoker		≥60 years	0.37	0.17–0.82	0.014
**Animal contact**	Logistic				
Yes vs no		14-29 years	2.35	1.34–4.12	0.003
**Time to hospital evaluation**	Multinomial				
8–21 days vs ≤7 days		14-29 years	0.62	0.40–0.96	0.034
>21 days vs ≤7 days		14-29 years	0.27	0.08–0.93	0.037
**Hospital admission**	Logistic				
Yes vs no		≥60 years	2.15	1.21–3.80	0.009
**ALT** ****	Multinomial				
2–10 × ULN vs normal		≥60 years	0.26	0.12–0.55	< 0.001
**GGT********	Logistic				
Elevated vs normal		14-29 years	0.43	0.27–0.68	< 0.001
**Diagnostic group**	Multinomial				
Highly probable vs confirmed		14-29 years	0.58	0.37–0.91	0.016
Highly probable vs confirmed		≥60 years	1.86	1.05–3.28	0.033
**Clinical group**	Multinomial				
Non specific vs hepatic		≥60 years	3.05	1.46–6.37	0.003
Pulmonary vs hepatic		≥60 years	7.80	3.06–19.87	< 0.001

* Only variables showing a statistically significant associations are included.
** The reference age group was 30–59 years.
*** OR: Odds ratio, 95% CI: 95% confidence interval.
**** ALT: alanine aminotransferase; GGT: gamma-glutamyl transferase, ULN: upper limit of normal.

Table S6a. Bivariate analysis of associations with time to clinical evaluation*

Variable	χ2	df**	p	Cramer's V
Platelets	51.42	4	< 0.001	0.28
aPTTr***	20.51	2	< 0.001	0.22
ESR***	16.27	2	< 0.001	0.21
CK***	6.91	2	0.032	0.15
ALT***	28.20	6	< 0.001	0.16
LDH***	17.70	2	< 0.001	0.21
GGT***	9.06	2	0.011	0.14
Diagnostic group	19.22	4	< 0.001	0.13
Clinical group	19.45	6	0.003	0.13

* Only variables showing a statistically significant association are included.
** df: degrees of freedom.
*** aPTT: activated partial thromboplastin time ratio; ESR: erythrocyte sedimentation rate; CK: creatine kinase; ALT: alanine aminotransferase; LDH: lactate dehydrogenase; GGT: gamma-glutamyl transferase.

Table S6b. Multivariate analysis of associations with time to clinical evaluation*

**Variable**	**Regression model**	**Comparison****	**OR*****	**95% CI****	**p value**
**Platelets**	Multinomial				
Thrombocytosis vs normal		> 21 days	4.38	1.87–10.26	< 0.001
Thrombocytopenia vs normal		≤ 7 days	2.46	1.59–3.80	< 0.001
**aPTTr********	Logistic				
Increased vs normal		≤ 7 days	0.43	0.28–0.66	< 0.001
**ESR********	Logistic				
Increased vs normal		≤ 7 days	0.37	0.23–0.61	< 0.001
**CK********	Logistic				
Increased vs normal		≤ 7 days	2.52	1.10–5.77	0.029
**ALT** ****	Multinomial				
1–2 × ULN vs normal		> 21 days	0.29	0.12–0.71	0.006
2–10 × ULN vs normal		> 21 days	0.11	0.04–0.31	< 0.001
**LDH** ****	Logistic				
Increased vs normal		> 21 days	0.20	0.08–0.47	< 0.001
**GGT** ****	Logistic				
Increased vs normal		≤ 7 days	0.55	0.36–0.83	0.005
**Diagnostic group**	Multinomial				
Highly probable vs confirmed		> 21 days	3.16	1.49–6.69	0.003
**Clinical group**	Multinomial				
Non specific vs hepatic		> 21 days	3.82	1.60–9.08	0.003
OLF**** vs hepatic		> 21 days	4.62	1.19–17.93	0.027

* Only variables showing statistically significant associations are included.
** The reference time category was 8-21 days.
*** OR: Odds ratio, 95% CI: 95% confidence interval.
**** aPTTr: Activated Partial Thromboplastin Time Ratio; ALT: Alanine aminotransferase; CK: Creatine Kinase; ESR: Erythrocyte Sedimentation Rate; GGT: Gamma-Glutamyl Transferase; LDH: Lactate Dehydrogenase; OLF: Other localized forms; ULN: Upper Limit of Normal.

Table S7a. Bivariate analysis of associations with clinical group*

Variable	$\chi 2$	df**	p	Cramer's V
Gender	18.50	3	< 0.001	0.18
Tobacco use	13.19	4	0.040	0.12
Alcohol use	13.04	4	0.042	0.12
Time to hospital evaluation	19.45	4	0.003	0.13
Hospital admission	87.24	2	< 0.001	0.39
Arthralgias	10.03	2	0.018	0.13
Headache	16.99	2	< 0.001	0.17
Myalgias	13.09	2	0.004	0.15
White blood cell count	65.93	4	< 0.001	0.24
Platelet count	34.72	4	< 0.001	0.17
C-reactive protein	9.49	2	0.023	0.18
ALT***	485.06	6	< 0.001	0.54
LDH***	70.44	2	< 0.001	0.40
GGT***	70.04	2	< 0.001	0.37
Overall urinalysis	17.79	2	< 0.001	0.21
Diagnostic group	55.96	2	< 0.001	0.22

* Only variables showing a statistically significant association are included.
** df: degrees of freedom.
*** ALT:alanine aminotransferase; LDH:lactate dehydrogenase; GGT:gamma-glutamyl transferase.

Table S7b. Multivariate analysis of associations with clinical group*

Variable	Regression model	Comparison**	OR***	95% CI***	p value
**Tobacco use**	Multinomial				
Former smoker vs non-smoker		Pulmonary	3.20	1.14–9.00	0.028
Current smoker vs non-smoker		Pulmonary	2.46	1.02–5.90	0.045
**Alcohol use**	Multinomial				
Excessive vs low/none		Pulmonary	8.44	2.01–35.49	0.004
**Time to hospital evaluation**	Multinomial				
8–21 days vs ≤7 days		Hepatic	0.26	0.11–0.62	0.002
**Hospital admission**	Logistic				
Yes vs no		Pulmonary	11.79	5-71–24.37	< 0.001
		Other localized form	8.19	4.09–16.40	< 0.001
**White blood cell count**	Multinomial				
Leukocytosis vs normal		Other localized form	4.27	2.04–8.96	< 0.001
Leukocytosis vs normal		Pulmonary	7.31	3.49–15.27	< 0.001
Leukopenia vs normal		Hepatic	3.80	1.81–7.99	< 0.001
**Platelet count**	Multinomial				
Thrombocytopenia vs normal		Hepatic	2.14	1.39–3.32	< 0.001
Thrombocytosis vs normal		Pulmonary	3.08	1.45–6.55	0.003
**C-reactive protein**	Logistic				
Increased vs normal		Hepatic	2.74	1.37–5.48	0.004
**LDH******	Logistic				
Increased vs normal		Hepatic	2.83	1.67–4.77	< 0.001
**GGT******	Logistic				
Increased vs normal		Hepatic	6.98	4.27–11.41	< 0.001
Increased vs normal		Other localized form	2.70	1.26–5.78	0.011
**Overall urinalysis**	Logistic				
Abnormal vs normal		Hepatic	3.15	1.74–5.73	< 0.001
**Diagnostic group**	Multinomial				
Highly probable vs confirmed		Hepatic	0.40	0.28–0.59	< 0.001
Highly probable vs confirmed		Other localized form	2.72	1.33–5.56	0.006
Highly probable vs confirmed		Pulmonary	2.81	1.38–5.73	0.005

* Only variables showing a statistically significant associations are included.
** The reference clinical group was "non specific".
*** OR: Odds ratio, 95% CI: 95% confidence interval.
**** GGT: gamma-glutamyl transferase; LDH:lactate dehydrogenase.
